# Peptide-based immunotherapy in lupus: Where are we now?

**DOI:** 10.2478/rir-2023-0020

**Published:** 2023-09-27

**Authors:** Ram P. Singh, David S. Bischoff, Satendra S Singh, Bevra H. Hahn

**Affiliations:** Research Service, Veteran Administration Greater Los Angeles Healthcare System, Los Angeles, 90073 CA, USA; Department of Medicine, University of California, Los Angeles, Los Angeles, 90095 CA, USA; Cedars-Sinai Medical Center, Beverly Hills, 90048 CA, USA

**Keywords:** peptide, immune tolerance, lupus, regulatory T cells, immunotherapy, cytokine, chemokine

## Abstract

In autoimmune rheumatic diseases, immune hyperactivity and chronic inflammation associate with immune dysregulation and the breakdown of immune self-tolerance. A continued, unresolved imbalance between effector and regulatory immune responses further exacerbates inflammation that ultimately causes tissue and organ damage. Many treatment modalities have been developed to restore the immune tolerance and immmunoregulatory balance in autoimmune rheumatic diseases, including the use of peptide-based therapeutics or the use of nanoparticles-based nanotechnology. This review summarizes the state-of-the-art therapeutic use of peptide-based therapies in autoimmune rheumatic diseases, with a specific focus on lupus.

## Introduction

Autoimmune rheumatic diseases carry a significant burden of morbidity and mortality and have a substantial economic impact on both individuals and the society as a whole.^[1, 2, 3, 4]^ Despite extensive research, the precise cellular and molecular mechanisms that drive the pathogenesis of those conditions are only partly understood, with a resulting variety of therapeutic approaches that depend on the symptoms, signs, and stage of the disease.

In systemic lupus erythematosus (SLE), corticosteroids are widely used to manage inflammation including in lupus nephritis, yet they carry significant side effects.^[5]^ Other immunosuppressive agents, including disease-modifying anti-rheumatic drugs (DMARDs) and biological therapies have shown some efficacy in inducing long-term remission but carry the risk of adverse events including the development of malignancy.^[6,7]^ In addition, treatments with biologics can be costly and increase the risk of systemic immune suppression.^[8]^

A safe alternative to the use of drugs carrying important side effects has been the use of peptide-based tolerogenic therapeutics for the induction of immune tolerance to self-antigens though the inhibition of autoreactive T, B, and dendritic cells together with the promotion of the activity of immunoregulatory cell populations.^[9, 10, 11]^ We discuss below the promise of peptide-based immunotherapies and related clinical trials.

## Peptide-Based Therapeutics

Peptide-based therapeutics can be broadly categorized into two categories that are based on their chief mechanism of action: immunogenic or tolerogenic. Immunogenic peptide therapy induces an immune response against a specific antigen, whereas the tolerogenic peptide therapy induces immune tolerance to self-antigens.

Immunogenic peptide therapy aims to activate the immune system to a specific antigen by presenting it to the immune cells in a way that mimics a natural infection.^[12, 13, 14]^ Although this approach has mainly been used in cancer immunotherapy to activate cytotoxic T lymphocytes (CTLs) against tumor antigens, several immunogenic peptide therapeutics have been developed as well for autoimmune diseases including SLE,^[15,16]^ rheumatoid arthritis,^[17,18]^ type 1 diabetes,^[19,20]^ and multiple sclerosis,^[21]^ with varying success.

Tolerogenic peptide therapy aims instead to induce immune tolerance to self-antigens by selectively targeting and suppressing self-reactive T cells, while promoting the expansion of regulatory T cells (T_regs_) and tolerogenic dendritic cells (DCs).^[22, 23, 24, 25, 26, 27, 28]^ This approach has been tested in several autoimmune diseases, including SLE, rheumatoid arthritis and multiple sclerosis. Tolerogenic peptide therapy can be further subcategorized according to the type of peptide(s) used, specifically:

(1) Epitope-specific peptides: These peptides are derived from specific epitopes of self-antigens and are used to selectively target autoreactive T cells.^[29, 30, 31]^ They can be delivered alone or in combination with immunomodulatory agents.

(2) Whole protein peptides: These peptides are derived from full-length self-proteins and can induce a broader immune response against multiple epitopes of the protein. They can be used to induce immune tolerance or to modulate the immune response in autoimmune diseases.^[13,32,33]^

(3) Mimotopes: They are typically a series of linear overlapping peptides designed to pinpoint either T cell or antibody epitopes and can be used to induce immune tolerance and/or to activate regulatory immune cells.^[34, 35, 36]^

## Strategies for Peptide-Based Therapy

A central characteristic of autoimmune diseases is the breakdown of self-tolerance with the activation of autoreactive immune cells that secrete pro-inflammatory cytokines and chemokines that amplify inflammation.

Two different strategies have been proposed to target these key aspects of the pathogenesis of autoimmunity^[37]^ (Table 1).

**Table 1:** Comparison of two peptide-based therapeutic strategies in autoimmune diseases including SLE

Comparative items	Peptide-based active therapy against proinflammatory cytokines	Peptide-based tolerogenic therapy
Source of peptides	Pro-inflammatory cytokines	Self-antigens, TCRs
Therapeutic targets	Pro-inflammatory cytokines	Autoimmune attacks against host cells or tissues
Main effects	Induced production of neutralizing antibodies	Immune tolerance to self-antigens through the inhibition of autoreactive lymphocytes and induction of T_regs_
Adjuvant	Adjuvant required	Not necessary
Relevant immune cells	Mainly B cells	Autoreactive T cells, T_regs_ (CD4^+^ and CD8^+^), tol DCs and other APCs
Evidence from clinical trials	Limited	Limited
Diseases	SLE, RA, SjS	SLE, RA, SjS

T_regs_: regulatory T cells; tol DCs: tolerogenic dendritic cells; RA: rheumatoid arthritis; SLE: systemic lupus erythematosus; SjS: Sjögren’s syndrome; TCRs: T cell receptors; APC: antigen presenting cells.

The first strategy aims to antagonize proinflammatory cytokines and chemokines that induce and sustain inflammation and favor the development of tissue damage.^[38, 39, 40, 41]^ This approach produces neutralizing antibodies (nAbs) against these proinflammatory biomolecules to inhibit their activities. In this strategy, the target immune cells are mostly B cells.

**Figure 1:**
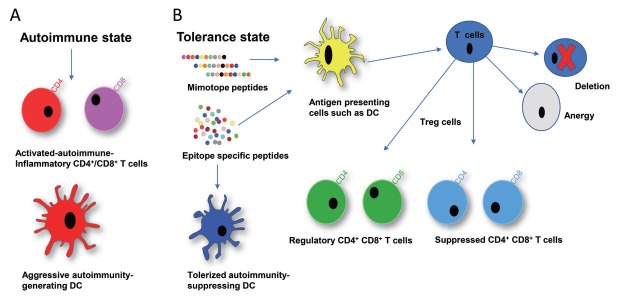
The role of peptide induced immune tolerance in SLE. A: In the autoimmune state, the CD4^+^ and CD8^+^ T cells are activated, and Dendritic cells (DCs) become aggressive and create autoimmune activation and further inflammation in SLE. B: In the tolerance state, peptide-induced tolerization promotes the expansion of antigen-specific regulatory cells and further tolerizes dendritic cells (DCs). These tolerized dendritic cells induce deletion and anergy of cognate T cells and further modulate regulatory T cells in SLE. These peptide induced regulatory T cells (T_regs_) which suppress the autoreactive/autoimmune responses in SLE.

The second strategy involves the development of peptide-based tolerogenic molecules that aim to restore immune homeostasis and rebalance immune dysregulation.^[9,10,22,24,25,27,28,42, 43, 44, 45, 46]^ The target immune cells in this strategy are autoreactive T cells, regulatory T cells (T_regs_), and tolerogenic dendritic cells (tol DCs), as schematized in Figure 1 for SLE.

Although peptide-based therapeutic approaches have shown promising results in inducing immune tolerance in autoimmune diseases, an optimization of the design and delivery of the peptides is still crucial to improve clinical efficacy.^[47]^

## Peptide-based Therapy against Pro-inflammatory Cytokines/Chemokines

Since pro-inflammatory cytokines and chemokines play a critical role in the pathogenesis of many autoimmune diseases, peptide-based vaccines have been designed to antagonize the pro-pathogenic cytokines and chemokines to reduce inflammation and tissue damage.

Modified peptides can target pro-inflammatory cytokines or chemokine receptors selectively. For example, a modified DNA peptide therapy targeting pro-inflammatory interleukin (IL)-17 significantly reduced organ damage and increased the survival of treated lupus-prone mice,^[48]^ while targeting the CXC chemokine ligand 10 (CXCL10) attenuated experimental autoimmune encephalomyelitis.^[49]^ Of interest, a peptide therapy targeting pro-inflammatory tumor necrosis factor alpha (TNF-α) induced the generation of T_regs_ and reduced inflammation in a mouse model of collagen induced arthritis.^[50,51]^

Potential biologics have been generated to target pro-inflammatory cytokines, such as IL-1, interferon (IFN)-α, IFN-γ, IL-6, TNF-α, IL-17, and IL-21, and several clinical trials that aim at inhibiting the activities of these mediators have been initiated.^[52]^ Despite the promising results in preclinical studies, there are still challenges that need to be addressed before translation into the clinic, including the identification of the optimal peptide targets and delivery methods, as well as the potential off-target effects and long-term safety.

## Peptide-Based Tolerogenic Therapeutics in Lupus

Recent studies both *in vitro* and *in vivo* have identified several peptides that are useful in lupus treatment. Table 2 indicates effects of the current peptide therapeutics in lupus.

**Table 2:** Salient features and effects of the current peptide therapeutics in SLE

Peptide	Origin	*In Vivo* Effects	Molecular Mechanisms	Administration Route	Clinical trial
pCons	Synthetic murine anti-dsDNA Ab	Reduced anti-DNA Ab, proteinuria; delayed nephritis and prolonged survival	Induces T_regs_ (both CD4^+^ and CD8^+^), increases TGF-b, IL-10, FOXP3	Intravenous route	N/A
hCDR1 (Edratide)	CDR1 of the human anti-DNA mAb	Decreased anti-dsDNA, anti-nuclear, anti-cardiolipin Abs, and nephritis; prolonged survival	Decreased IFN- γ, IFN- α, IL-1B, TNF-α, BAFF, caspases 3 and 8, T and B cell activation, Increased CD4^+^ and CD8^+^ T_regs_, and B cell apoptosis	Subcutaneous	Double blind, Phase 2 placebo-controlled clinical trial
DWEYS	Anti-DNA Ab (D/E W D/E Y S/G), consensus sequence with NMDAR	Decreased anti-dsDNA Abs; amelioration of renal and CNS manifestations	Decreased binding and glomerular deposition of anti dsDNA Abs; inhibition of autoreactive B cells	Intravenous route	N/A
FISLE-412	Molecular topology of DWEYS	Decreased anti-dsDNA Abs, amelioration of renal and CNS manifestations	Decreased binding and glomerular deposition of anti dsDNA Abs, Abs to cardiolipin, and neuronal apoptosis	Oral	N/A
ALW	Four types of murine anti-dsDNA IgG mAbs	Decreased anti-dsDNA Abs; amelioration of renal manifestations	Decreased laminin, CTGF, TGF-b, PDGF-B, anti-DNA Ab binding/glomerular deposition; renal inflammation	Intravenous route	N/A
Nucleo-somal histone peptide	Endogenous peptide epitopes in histone from nucleosome	Decreased pathogenic autoantibody production, renal inflammation, B cell activation	Induced both CD4^+^ and CD8^+^ T_regs_	Subcutaneous intranasal, intraperitoneal	N/A
LJP-394 (Abetimus)	Synthetic tolerogen (4 oligo-nucleotides attached to niPEG)	Decreased anti-dsDNA Abs	Binds to cross-linking surface Abs	Intravenous	Phase I, II and III

SLE, systemic lupus erythematosus; TGF-β, transforming growth factor β; FOXP3, Forkhead box protein P3; SOCS-1 suppression of cytokine signaling-1, PDGF-B, platelet-derived growth factor-B; CTGF, connective tissue growth factor; CNS, central nervous system; Abs, antibodies; niPEG, non-immunogenic polyethylene glycol; N/A, not available. Modified from ref [86], Springer.

### pCons Peptide

pCons (pConsensus-FIEWNKLRFRQGLEW) is a 15-mer peptide, derived from the V^H^ regions of anti-DNA antibodies from (NZB x NZW) F1 (BWF1) mice.^[9,10,22,27,28,43,53]^ pCons peptide induce immune tolerance therapy in BWF1 lupus-mice by promoting the generation of autoantigen-specific regulatory T cells that suppressed effector T cells^[11,54]^ and autoantibody-producing B cells.^[55]^

pCons peptide therapy induces immune tolerance by promoting expansion of CD8^+^ and CD4^+^ T_regs._^[9,10,22,25,27,28,53,56]^ pCons-induced T_regs_ suppress the production of anti-dsDNA autoantibodies, reduce proteinuria, and delay nephritis in murine lupus.^[22,53]^ Notably, oral administration of different forms of pCons peptide, including D and L forms, delayed anti-ds-DNA Ab production and lupus nephritis, and increased the survival of lupus mice.^[57]^

Importantly, pCons peptide induced T_regs_ in lupus patients.^[58]^ When the lupus patients’ peripheral blood mononuclear cells (PBMCs) were cultured with pCons peptide, the expansion of fully functional T_regs_ occurred in those patients that were seropositive for anti-DNA autoantibodies. This is in line with the antigen-specificity of the response promoted by peptide immunotherapy, and resulted in the suppression by the pCons-induced T_regs_ of the proliferation of CD4^+^CD25^-^ T effectors cells and reduced production of proinflammatory cytokines.^[58]^ Clinical trials are awaited to address whether the promising effects of pCons can be translated into clinical settings.

### human complementarity-determing region 1 (hCDR1) (Edratide)

hCDR1 (edratide) is a 19-mer peptide based on the heavy chain complementarity-determining region one (CDR1) sequences of a human anti-DNA monoclonal antibody. Edratide reduced autoreactive T cells responses by inducing T _regs_ and by downregulating the production of IFN-α in both murine and human SLE.^[25,59, 60, 61]^ Tolerization with hCDR1 induced both CD4^+^CD25^+^ and CD8^+^CD28^−^ T_regs_ in mice, which suppressed lymphocyte proliferation and autoantibody production.^[62, 63, 64]^ Earlier studies had shown that hCDR1 induced anergy in antigen presenting cells (APCs) and the generation of Tr1 T_regs_, which expressed tolerance-associated genes.^[24,59,60]^ hCDR1 also downregulated IL-1β, IFN-γ, and TNF-α in addition to IFN-α, and upregulated the immunosuppressive cytokine transforming growth factor-β (TGF-β). It also reduced expression of Lymphocyte function-associated antigen 1 (LFA-1) and CD44 and regulated Jun-N terminal kinase (JNK) in the reduction of the apoptosis of T cells.^[65,66]^ hCDR1 treatment delayed the production of both anti-DNA antibodies and proteinuria, and increased the survival of lupus-prone mice by regulating B cell activating factor (BAFF).^[67]^ Additionally, hCDR1 improved cognitive behavior and brain pathology in lupus mice^[68]^ and downregulated the expression of the indoleamine 2, 3-dioxygenase (IDO) gene, which is increased in SLE patients.^[47]^

A phase II clinical trial with hCDR1 (Edratide) was conducted in 340 SLE patients and demonstrated that it was safe and well-tolerated. However, the co-primary endpoints of a reduction of SLE disease activity index (SLEDAI-2K) and adjusted mean SLEDAI (AMS) in patients compared with controls using a landmark analysis were not met.^[69]^ Secondary outcomes were improvement in the British Isles Lupus Assessment Group (BILAG) Responder Index and medicinal flare analysis. There was a positive trend in the Composite SLE Responder Index in the Intention-to-treat (ITT) cohort, and post-hoc analysis showed that the BILAG secondary endpoint was met for a number of subgroups dosing levels, including low or no steroids, seropositivity, and patients with 2 grade BILAG improvement.

### Nucleosomal Histone Peptides

Endogenous peptide epitopes of histones from nucleosomes induce immune tolerance and promote CD4^+^ and CD8^+^ T_reg_ responses, and reduce lupus nephritis and B cell activation in (SWR x NZB) F1 (SNF1) mice,^[70, 71, 72]^ also blocking pathogenic autoimmunity in human SLE.^[73,74]^

Other peptides derived from histone proteins have shown similar effects and have been investigated as therapeutic candidates in SLE,^[75, 76, 77]^ including the phosphorylated spliceosomal epitope peptide P140, which represses B cell differentiation and protects against murine lupus.^[78, 79, 80]^ Encouraging clinical trials indicate that it may improve clinical parameters in SLE patients.^[15,81,82]^

### DWEYS Peptide

DWEYS ((D/EWD/EYS/G)) peptide was identified using the mouse monoclonal anti-dsDNA R4A antibody.^[83, 84, 85, 86]^ It inhibits the R4A mAb from binding to dsDNA in animal models of lupus and SLE patients. It has been shown that the nephritogenic mouse monoclonal antibody R4A binds to a consensus pentapeptide sequence D/EWD/EYS/G (DWEYS), present in both NR2A and NR2B subunits of the mouse and human N-methyl-d-aspartate receptor (NMDAR).^[84,85,87]^ Anti-DWEYS and anti-dsDNA antibodies have cross-reactivity with NMDAR.^[83]^ Intravenous administration of the DWEYS peptide to gestating mice had protective effects on the fetal brains when exposed to toxic doses of anti-NMDAR antibodies.^[88]^ In *ex vivo* studies in lupus patients with anti-DNA antibodies and active lupus nephritis, the DWEYS peptide inhibited DNA binding.^[88, 89, 90]^ Furthermore, the peptide suppressed human autoreactive B cells and the production of anti-dsDNA antibodies *in vitro*.^[90]^

### FISLE-412

FISLE-412 is a small molecule peptidomimetic that can neutralize anti-dsDNA autoantibodies associated with SLE. FISLE-412 has shown effectiveness *in vitro* and *in vivo*.^[91, 92, 93]^ It inhibits lupus autoantibody-mediated antigen recognition by human SLE patient serum and further prevents pathogenic interactions with tissue antigens.^[91]^ In addition, it suppresses pathogenic deposition of SLE autoantibodies into the kidney glomeruli, and blocks neurotoxicity *in vivo*.^[91]^ Based on its structure-activity relationships, several analogues of FISLE-412 have been developed and found to neutralize anti-lupus antibodies *in vitro* and *ex vivo*.^[92]^ FISLE-412 is well-tolerated and not immunogenic; as well as being stable, easily synthesized in high-throughput, and inexpensive to produce.^[92,94]^

### ALW

ALW (ALWPPNLHAWVP) is a 12-mer peptide mimic identified from the four different types of murine monoclonal anti-DNA immunoglobulin G (IgG, including IgG1, IgG2a, IgG2b, and IgG3) by screening phage display libraries.^[95]^ ALW peptide exhibits isotype-dependent binding to anti-DNA antibodies in a sequence-specific manner. In addition, it markedly inhibits the binding of anti-DNA antibodies in human and mouse lupus sera to dsDNA and to laminin self-antigen. A protective effect of ALW peptide was demonstrated in MRL lymphoproliferation strain lupus mice, where it inhibited glomerular deposition of antibodies, reduced serum-anti-dsDNA antibodies, and improved overall renal pathology.^[96]^ Due to the absence of amino acids such as methionine, cysteine, and glutamine, ALW is physiologically stable and is resistant to peptide oxidation, cyclization, and degradation.^[97]^ It is nontoxic, non-immunogenic, and has a net neutral charge that reduces non-specific protein-protein interactions. As such, ALW or its analogues are seen as good potential novel candidate therapeutics in SLE.

### LJP-394 (Abetimus Sodium)

LJP-394 (Abetimus sodium) is a synthetic peptide developed to induce immune tolerance to double-stranded DNA (dsDNA), a key autoantigen in SLE.^[98]^ It is a tetrameric oligonucleotide conjugate that can reduce anti-dsDNA antibody levels, minimize nephritic flares,^[98]^ and act as a B cell tolerogen.^[99]^ It has been suggested that the relative affinity of dsDNA antibodies from SLE patients for LJP-394 may impact their responses to the drug, and patients with high-affinity antibodies to LJP-394 experience a reduction in affinity after four months of weekly treatment.^[100]^

LJP-394 has been evaluated in 14 clinical trials^[101]^ and has shown to be safe and reduce circulating anti-dsDNA antibodies and disease activity in patients with active SLE. However, two pivotal trials failed to meet the primary endpoint of a statistically significant prolongation in the time to renal flare.^[98,102]^ In another clinical trial, a dosage of 100 mg/week of LJP-394 significantly reduced anti-dsDNA antibody levels but did not significantly prolong the time to renal flare compared to placebo. Nonetheless, several positive trends in endpoints were observed in the treated group,^[103]^ leading to wonder how to improve targeting of this agent to the most suitable SLE patients.

## Use of Nanotechnology in Peptide-Induced Tolerance in SLE

Nanotechnology is a promising field for the development of new therapeutics including in autoimmune rheumatic diseases.^[104, 105, 106]^ In SLE, nanotechnology-based strategies have been developed to induce immune tolerance to self-antigens^[107]^ and to deliver peptides that modulate the immune response.^[108]^

Nanoparticles offers several advantages over traditional drug delivery systems, including improved pharmacokinetics, enhanced stability, and reduced toxicity.^[105]^ In SLE, nanoparticles have been used to deliver lupus-specific peptides, selectively targeting autoimmune B cells, and inducing immune tolerance. For instance, poly-(lactic-co-glycolic acid) (PLGA) nanoparticles loaded with a lupus-specific peptide (P140) have demonstrated efficacy in reducing autoantibody production and renal damage in lupus-prone mice.^[109]^ Another study utilized T cell targeted PLGA-nanoparticles encapsulating IL-2 and TGF-β resulting in the expansion of both CD4^+^ and CD8^+^ T_regs_ *in vivo* and suppression of murine lupus.^[110]^ Additionally, the delivery of a calcium/calmodulin-dependent protein kinase-IV (CAMK4) inhibitor via nanoparticles ameliorated murine SLE.^[111,112]^ Furthermore, microRNA125a-loaded nanoparticles have also been found to restore homeostasis and ameliorate murine lupus by regulating the balance between T effector cells and T_regs._^[113]^

Apart from delivering lupus-specific peptides, nanoparticles can be engineered to target specific immune cells and tissues. For example, mesoporous silica nanoparticles (MSNs) functionalized with lupus-specific peptides and antibodies have been developed to selectively target immune cells and tissues.^[114, 115, 116, 117]^ Similarly, magnetic nanoparticles (MNPs) coated with a lupus-specific peptide have been shown to selectively target lupus B cells and induce immune tolerance.^[110,118,119]^ Furthermore, nanotechnology-based strategies enable the delivery of immunomodulatory agents such as immunosuppressants to target specific immune cells and tissues. For instance, PLGA nanoparticles loaded with rapamycin, an immunosuppressant, have shown effectiveness in reducing autoantibody production and renal damage in lupus-prone mice.^[120,121]^ Also, dendrimers loaded with anti-inflammatory IL-10 reduce disease activity in mice.^[122, 123, 124]^

## Conclusions and Future Perspectives

Peptide-based therapies for lupus have shown great promise in preclinical but clinical studies have been so far rather limited. This mandates additional clinical studies to better elucidate the potential of peptide-based therapies in SLE. In clinical studies, peptides have demonstrated safety and tolerability; however, only variable efficacy has been demonstrated in reducing disease activity in SLE patients, possibly due to trial design that cannot preselect those patients that might benefit the most from peptide immunotherapy. Of note, peptides have nonetheless consistently shown the potential to induce immune tolerance, which is dysregulated in SLE patients. Peptide-based therapies have also shown the potential to selectively target specific immune cell populations, either B cell or T cell populations.

Peptides such as ALW and FISLE-412 have demonstrated the ability to neutralize lupus autoantibodies and prevent their pathogenic interactions with tissue antigens. More recently, the encapsulation of peptides in nanocarriers is leading to agents that can have improved pharmacokinetics, biodistri-bution, and bioavailability, as well as enhanced targe specificity. Nanoparticles can also help to reduce toxicity.

Despite this all, there are still challenges that need to be addressed. One is identifying the optimal peptide sequences and dosage to achieve the most effective outcomes. Another challenge is to develop the proper biomarkers to monitor response and predict treatment outcomes. One important consideration is that combination therapies that involve peptides and other immunomodulatory agents can developed but have so far not been tested.

To conclude, peptide-based therapies can promote immune tolerance and have shown great potential in reducing disease activity in lupus patients. With further research, new technologies such as nanotechnology and more information on how to better refine what we know for better clinical trials, peptide-based therapies could become a new avenue of treatment for SLE.

## References

[1] Bultink IEM, de Vries F, van Vollenhoven RF (2021). Mortality, causes of death and influence of medication use in patients with systemic lupus erythematosus vs matched controls. Rheumatology (Oxford).

[2] Lau CS, Mak A (2009). The socioeconomic burden of SLE. Nat Rev Rheumatol.

[3] Løppenthin K, Esbensen BA, Østergaard M (2019). Morbidity and mortality in patients with rheumatoid arthritis compared with an age- and sex-matched control population: A nationwide register study. J Comorb.

[4] (2011). Tsokos GC. Systemic lupus erythematosus. N Engl J Med.

[5] Guillermo Ruiz-Irastorza, George Bertsias (2020). Treating systemic lupus erythematosus in the 21st century. new drugs and new perspectives on old drugs. Rheumatology (Oxford).

[6] Broen JCA, van Laar JM (2020). Mycophenolate mofetil, azathioprine and tacrolimus: mechanisms in rheumatology. Nat Rev Rheumatol.

[7] Burmester GR, Pope JE (2017). Novel treatment strategies in rheumatoid arthritis. Lancet.

[8] Williams EL, Gadola S, Edwards CJ (2009). Anti-TNF-induced lupus. Rheumatology (Oxford).

[9] Singh RP, Hahn BH, Bischoff DS (2021). Effects of Peptide-Induced Immune Tolerance on Murine Lupus. Front Immunol.

[10] Singh RP, Hahn BH, Bischoff DS. Cellular, Molecular Phenotypes (2021). of pConsensus Peptide (pCons) Induced CD8+ and CD4+ Regulatory T Cells in Lupus. Front Immunol.

[11] Yu Y, Liu Y, Shi FD (2012). Tolerance induced by anti-DNA Ig peptide in (NZB×NZW)F1 lupus mice impinges on the resistance of effector T cells to suppression by regulatory T cells. Clin Immunol.

[12] Black M, Trent A, Tirrell M (2010). Advances in the design and delivery of peptide subunit vaccines with a focus on toll-like receptor agonists. Expert Rev Vaccines.

[13] Gokhale AS, Satyanarayanajois S. Peptides (2014). peptidomimetics as immunomodulators. Immunotherapy.

[14] Shoenfeld Y (2005). Anti-DNA idiotypes: from induction of disease to novel therapeutical approaches. Immunol Lett.

[15] Schall N, Muller S (2015). Resetting the autoreactive immune system with a therapeutic peptide in lupus. Lupus.

[16] Favoino E, Prete M, Marzullo A (2017). CD20-Mimotope Peptide Active Immunotherapy in Systemic Lupus Erythematosus and a Reappraisal of Vaccination Strategies in Rheumatic Diseases. Clin Rev Allergy Immunol.

[17] Rosenthal KS, Mikecz K (2015). Rheumatoid arthritis vaccine therapies: perspectives and lessons from therapeutic ligand epitope antigen presentation system vaccines for models of rheumatoid arthritis. Expert Rev Vaccines.

[18] Mikecz K, Glant TT, Markovics A (2017). An epitope-specific DerG-PG70 LEAPS vaccine modulates T cell responses and suppresses arthritis progression in two related murine models of rheumatoid arthritis. Vaccine.

[19] Smith EL, Peakman M (2018). Peptide Immunotherapy for Type 1 Diabetes-Clinical Advances. Front Immunol.

[20] Arif S, Gomez-Tourino I, Kamra Y (2020). GAD-alum immunotherapy in type 1 diabetes expands bifunctional Th1/Th2 auto-reactive CD4 T cells. Diabetologia.

[21] Zorzella-Pezavento SFG, Mimura LAN, Fraga-Silva TFC (2017). Experimental Autoimmune Encephalomyelitis Is Successfully Controlled by Epicutaneous Administration of MOG Plus Vitamin D Analog. Front Immunol.

[22] Hahn BH, Singh RP, La Cava A (2005). Tolerogenic treatment of lupus mice with consensus peptide induces Foxp3-expressing, apoptosis-resistant, TGFbeta-secreting CD8+ T cell suppressors. J Immunol.

[23] La Cava A, Fang CJ, Singh RP (2005). Manipulation of immune regulation in systemic lupus erythematosus. Autoimmun Rev.

[24] Mozes E, Sharabi A (2010). A novel tolerogenic peptide, hCDR1, for the specific treatment of systemic lupus erythematosus. Autoimmun Rev.

[25] Sharabi A, Haviv A, Zinger H (2006). Amelioration of murine lupus by a peptide, based on the complementarity determining region-1 of an autoantibody as compared to dexamethasone: different effects on cytokines and apoptosis. Clin Immunol.

[26] Singh RP, Hahn BH, La Cava A (2008). Tuning immune suppression in systemic autoimmunity with self-derived peptides. Inflamm Allergy Drug Targets.

[27] Singh RP, La Cava A, Hahn BH (2008). pConsensus peptide induces tolerogenic CD8+ T cells in lupus-prone (NZB x NZW)F1 mice by differentially regulating Foxp3 and PD1 molecules. J Immunol.

[28] Singh RP, La Cava A, Wong M (2007). CD8+ T cell-mediated suppression of autoimmunity in a murine lupus model of peptide-induced immune tolerance depends on Foxp3 expression. J Immunol.

[29] Burkhardt H, Koller T, Engström A (2002). Epitope-specific recognition of type II collagen by rheumatoid arthritis antibodies is shared with recognition by antibodies that are arthritogenic in collagen-induced arthritis in the mouse. Arthritis Rheum.

[30] Daveson AJM, Ee HC, Andrews JM (2017). Epitope-Specific Immunotherapy Targeting CD4-Positive T Cells in Celiac Disease: Safety, Pharmacokinetics, and Effects on Intestinal Histology and Plasma Cytokines with Escalating Dose Regimens of Nexvax2 in a Randomized, Double-Blind, Placebo-Controlled Phase 1 Study. EBioMedicine.

[31] Zhao Z, Ren J, Dai C (2019). Nature of T cell epitopes in lupus antigens and HLA-DR determines autoantibody initiation and diversification. Ann Rheum Dis.

[32] Monneaux F, Muller S (2004). Peptide-based immunotherapy of systemic lupus erythematosus. Autoimmun Rev.

[33] Datta SK (2021). Harnessing Tolerogenic Histone Peptide Epitopes From Nucleosomes for Selective Down-Regulation of Pathogenic Autoimmune Response in Lupus (Past, Present, and Future). Front Immunol.

[34] Jürgen W Dieker, Yong-Jiang Sun, Cor W Jacobs (2005). Mimotopes for lupus-derived anti-DNA and nucleosome-specific autoantibodies selected from random peptide phage display libraries: facts and follies. J Immunol Methods.

[35] Sibille P, Ternynck T, Nato F (1997). Mimotopes of polyreactive anti-DNA antibodies identified using phage-display peptide libraries. Eur J Immunol.

[36] Slansky JE, Nakayama M (2020). Peptide mimotopes alter T cell function in cancer and autoimmunity. Semin Immunol.

[37] Wang B, Chen S, Zheng Q (2020). Peptide-Based Vaccination Therapy for Rheumatic Diseases. J Immunol Res.

[38] Dinarello CA (2010). Anti-inflammatory Agents: Present and Future. Cell.

[39] La Cava A (2013). Targeting the BLyS-APRIL signaling pathway in SLE. Clin Immunol.

[40] Semerano L, Assier E, Boissier MC (2012). Anti-cytokine vaccination: a new biotherapy of autoimmunity?. Autoimmun Rev.

[41] Zagury D, Burny A, Gallo RC (2001). Toward a new generation of vaccines: the anti-cytokine therapeutic vaccines. Proc Natl Acad Sci U S A.

[42] Amarilyo G, Hahn B, La Cava A (2012). Preclinical studies with synthetic peptides in systemic lupus erythematosus. Front Biosci (Landmark Ed).

[43] Hahn BH, Singh RR, Wong WK (2001). Treatment with a consensus peptide based on amino acid sequences in autoantibodies prevents T cell activation by autoantigens and delays disease onset in murine lupus. Arthritis Rheum.

[44] Schall N, Page N, Macri C (2012). Peptide-based approaches to treat lupus and other autoimmune diseases. J Autoimmun.

[45] Singh RR, Kumar V, Ebling FM (1995). T cell determinants from autoantibodies to DNA can upregulate autoimmunity in murine systemic lupus erythematosus. J Exp Med.

[46] Spiering R, Margry B, Keijzer C (2015). DEC205+ Dendritic Cell-Targeted Tolerogenic Vaccination Promotes Immune Tolerance in Experimental Autoimmune Arthritis. J Immunol.

[47] Sthoeger Z, Sharabi A, Zinger H (2018). Indoleamine-2,3-dioxygenase in murine and human systemic lupus erythematosus: Down-regulation by the tolerogeneic peptide hCDR1. Clin Immunol.

[48] Koriyama H, Ikeda Y, Nakagami H (2020). Development of an IL-17A DNA Vaccine to Treat Systemic Lupus Erythematosus in Mice. Vaccines (Basel).

[49] O’Connor RA, Li X, Blumerman S (2012). Adjuvant immunotherapy of experimental autoimmune encephalomyelitis: immature myeloid cells expressing CXCL10 and CXCL16 attract CXCR3+CXCR6+ and myelin-specific T cells to the draining lymph nodes rather than the central nervous system. J Immunol.

[50] Lamontain V, Schmid T, Weber-Steffens D (2019). Stimulation of TNF receptor type 2 expands regulatory T cells and ameliorates established collagen-induced arthritis in mice. Cell Mol Immunol.

[51] Mark Farrugia, Byron Baron (2016). The role of TNF-α in rheumatoid arthritis: a focus on regulatory T cells. J Clin Transl Res.

[52] Wong M, La Cava A (2011). Lupus, the current therapeutic approaches. Drugs Today (Barc).

[53] La Cava A, Ebling FM, Hahn BH (2004). Ig-reactive CD4+CD25+ T cells from tolerized (New Zealand Black x New Zealand White) F1 mice suppress in vitro production of antibodies to DNA. J Immunol.

[54] Iikuni N, Hahn BH, La Cava A (2009). Potential for anti-DNA immunoglobulin peptide therapy in systemic lupus erythematosus. Expert Opin Biol Ther.

[55] Iikuni N, Lourenço EV, Hahn BH (2009). Cutting edge: Regulatory T cells directly suppress B cells in systemic lupus erythematosus. J Immunol.

[56] Singh RR, Ebling FM, Sercarz EE (1995). Immune tolerance to autoantibody-derived peptides delays development of autoimmunity in murine lupus. J Clin Invest.

[57] Skaggs BJ, Singh RP, Hahn BH (2008). Induction of immune tolerance by activation of CD8+ T suppressor/regulatory cells in lupus-prone mice. Hum Immunol.

[58] Hahn BH, Anderson M, Le E (2008). Anti-DNA Ig peptides promote Treg cell activity in systemic lupus erythematosus patients. Arthritis Rheum.

[59] Sharabi A, Zinger H, Zborowsky M (2006). A peptide based on the complementarity-determining region 1 of an autoantibody ameliorates lupus by up-regulating CD4+CD25+ cells and TGF-beta. Proc Natl Acad Sci U S A.

[60] Sthoeger Z, Zinger H, Sharabi A (2013). The tolerogenic peptide, hCDR1, down-regulates the expression of interferon-α in murine and human systemic lupus erythematosus. PLoS One.

[61] Waisman A, Ruiz PJ, Israeli E (1997). Modulation of murine systemic lupus erythematosus with peptides based on complementarity determining regions of a pathogenic anti-DNA monoclonal antibody. Proc Natl Acad Sci U S A.

[62] Crispín JC, Kyttaris VC, Terhorst C (2010). T cells as therapeutic targets in SLE. Nat Rev Rheumatol.

[63] Dinesh RK, Skaggs BJ, La Cava A (2010). CD8+ Tregs in lupus, autoimmunity, and beyond. Autoimmun Rev.

[64] Horwitz DA, Zheng SG, Gray JD (2004). Regulatory T cells generated ex vivo as an approach for the therapy of autoimmune disease. Semin Immunol.

[65] Rapoport MJ, Sharabi A, Aharoni D (2005). Amelioration of SLE-like manifestations in (NZBxNZW)F1 mice following treatment with a peptide based on the complementarity determining region 1 of an autoantibody is associated with a down-regulation of apoptosis and of the pro-apoptotic factor JNK kinase. Clin Immunol.

[66] Sthoeger Z, Sharabi A, Mozes E (2014). Novel approaches to the development of targeted therapeutic agents for systemic lupus erythematosus. J Autoimmun.

[67] Parameswaran R, Ben David H, Sharabi A (2009). B-cell activating factor (BAFF) plays a role in the mechanism of action of a tolerogenic peptide that ameliorates lupus. Clin Immunol.

[68] Lapter S, Marom A, Meshorer A (2009). Amelioration of brain pathology and behavioral dysfunction in mice with lupus following treatment with a tolerogenic peptide. Arthritis Rheum.

[69] Urowitz MB, Isenberg DA, Wallace DJ (2015). Safety and efficacy of hCDR1 (Edratide) in patients with active systemic lupus erythematosus: results of phase II study. Lupus Sci Med.

[70] Kang HK, Michaels MA, Berner BR (2005). Very low-dose tolerance with nucleosomal peptides controls lupus and induces potent regulatory T cell subsets. J Immunol.

[71] Kang JY, Lee JO (2011). Structural biology of the Toll-like receptor family. Annu Rev Biochem.

[72] Kang SM, Tang Q, Bluestone JA (2007). CD4+CD25+ regulatory T cells in transplantation: progress, challenges and prospects. Am J Transplant.

[73] Zhang L, Bertucci AM, Ramsey-Goldman R (2009). Regulatory T cell (Treg) subsets return in patients with refractory lupus following stem cell transplantation, and TGF-beta-producing CD8+ Treg cells are associated with immunological remission of lupus. J Immunol.

[74] Zhang L, Bertucci AM, Ramsey-Goldman R (2013). Major pathogenic steps in human lupus can be effectively suppressed by nucleosomal histone peptide epitope-induced regulatory immunity. Clin Immunol.

[75] Shapira E, Proscura E, Brodsky B (2011). Novel peptides as potential treatment of systemic lupus erythematosus. Lupus.

[76] Wu HY, Staines NA (2004). A deficiency of CD4+CD25+ T cells permits the development of spontaneous lupus-like disease in mice, and can be reversed by induction of mucosal tolerance to histone peptide autoantigen. Lupus.

[77] Wu HY, Ward FJ, Staines NA (2002). Histone peptide-induced nasal tolerance: suppression of murine lupus. J Immunol.

[78] Monneaux F, Lozano JM, Patarroyo ME (2003). T cell recognition and therapeutic effect of a phosphorylated synthetic peptide of the 70K snRNP protein administered in MR/lpr mice. Eur J Immunol.

[79] Page N, Gros F, Schall N (2011). A therapeutic peptide in lupus alters autophagic processes and stability of MHCII molecules in MRL/lpr B cells. Autophagy.

[80] Wilhelm M, Wang F, Schall N (2018). Lupus Regulator Peptide P140 Represses B Cell Differentiation by Reducing HLA Class II Molecule Overexpression. Arthritis Rheumatol.

[81] Muller S, Monneaux F, Schall N (2008). Spliceosomal peptide P140 for immunotherapy of systemic lupus erythematosus: results of an early phase II clinical trial. Arthritis Rheum.

[82] Zimmer R, Scherbarth HR, Rillo OL (2013). Lupuzor/P140 peptide in patients with systemic lupus erythematosus: a randomised, double-blind, placebo-controlled phase IIb clinical trial. Ann Rheum Dis.

[83] Bosch X, Ramos-Casals M, Khamashta MA (2012). The DWEYS peptide in systemic lupus erythematosus. Trends Mol Med.

[84] DeGiorgio LA, Konstantinov KN, Lee SC (2001). A subset of lupus anti-DNA antibodies cross-reacts with the NR2 glutamate receptor in systemic lupus erythematosus. Nat Med.

[85] Gaynor B, Putterman C, Valadon P (1997). Peptide inhibition of glomerular deposition of an anti-DNA antibody. Proc Natl Acad Sci U S A.

[86] Wang Y, Xiao S, Xia Y (2022). The Therapeutic Strategies for SLE by Targeting Anti-dsDNA Antibodies. Clin Rev Allergy Immunol.

[87] Shefner R, Kleiner G, Turken A (1991). A novel class of anti-DNA antibodies identified in BALB/c mice. J Exp Med.

[88] Diamond B, Bloom O, Al Abed Y (2011). Moving towards a cure: blocking pathogenic antibodies in systemic lupus erythematosus. J Intern Med.

[89] Sharma A, Isenberg D, Diamond B (2003). Studies of human polyclonal and monoclonal antibodies binding to lupus autoantigens and cross-reactive antigens. Rheumatology (Oxford).

[90] Voynova E, Tchorbanov A, Prechl J (2008). An antibody-based construct carrying DNA-mimotope and targeting CR1(CD35) selectively suppresses human autoreactive B-lymphocytes. Immunol Lett.

[91] Bloom O, Cheng KF, He M (2011). Generation of a unique small molecule peptidomimetic that neutralizes lupus autoantibody activity. Proc Natl Acad Sci U S A.

[92] He M, Cheng KF, VanPatten S (2017). A structural investigation of FISLE-412, a peptidomimetic compound derived from saquinavir that targets lupus autoantibodies. Bioorg Med Chem Lett.

[93] VanPatten S, Sun S, He M (2016). Amending HIV Drugs: A Novel Small-Molecule Approach To Target Lupus Anti-DNA Antibodies. J Med Chem.

[94] Altiti AS, Cheng KF, He M (2017). β-Hydroxy-tetrahydroquinolines from Quinolines Using Chloroborane: Synthesis of the Peptidomimetic FISLE-412. Chemistry.

[95] Xia Y, Eryilmaz E, Der E (2016). A peptide mimic blocks the cross-reaction of anti-DNA antibodies with glomerular antigens. Clin Exp Immunol.

[96] Wang H, Lu M, Zhai S (2019). ALW peptide ameliorates lupus nephritis in MRL/lpr mice. Arthritis Res Ther.

[97] Sibylle Hoeppe, Thomas D Schreiber, Hannes Planatscher (2011). Targeting peptide termini, a novel immunoaffinity approach to reduce complexity in mass spectrometric protein identification. Mol Cell Proteomics.

[98] Horowitz DM, Furie RA (2009). Abetimus sodium: a medication for the prevention of lupus nephritis flares. Expert Opin Pharmacother.

[99] Wallace DJ, Tumlin JA (2004). LJP 394 (abetimus sodium, Riquent) in the management of systemic lupus erythematosus. Lupus.

[100] Lorenz HM (2002). Abetimus (La Jolla pharmaceuticals). Curr Opin Investig Drugs.

[101] Mosca M, Baldini C, Bombardieri S (2007). LJP-394 (abetimus sodium) in the treatment of systemic lupus erythematosus. Expert Opin Pharmacother.

[102] Richard Furie (2006). Abetimus sodium (riquent) for the prevention of nephritic flares in patients with systemic lupus erythematosus. Rheum Dis Clin North Am.

[103] Cardiel MH, Tumlin JA, Furie RA (2008). Abetimus sodium for renal flare in systemic lupus erythematosus: results of a randomized, controlled phase III trial. Arthritis Rheum.

[104] Gharagozloo M, Majewski S, Foldvari M (2015). Therapeutic applications of nanomedicine in autoimmune diseases: from immunosuppression to tolerance induction. Nanomedicine (Lond).

[105] Horwitz DA, Bickerton S, La Cava A (2021). Strategies to Use Nanoparticles to Generate CD4 and CD8 Regulatory T Cells for the Treatment of SLE and Other Autoimmune Diseases. Front Immunol.

[106] Yang Y, Santamaria P (2021). Evolution of nanomedicines for the treatment of autoimmune disease: From vehicles for drug delivery to inducers of bystander immunoregulation. Adv Drug Deliv Rev.

[107] Ferretti C, Horwitz DA, Bickerton S (2021). Nanoparticle-mediated Delivery of IL-2 To T Follicular Helper Cells Protects BDF1 Mice from Lupus-like Disease. Rheumatol Immunol Res.

[108] Li H, Yang YG, Sun T (2022). Nanoparticle-Based Drug Delivery Systems for Induction of Tolerance and Treatment of Autoimmune Diseases. Front Bioeng Biotechnol.

[109] Schall N, Talamini L, Wilhelm M (2022). P140 Peptide Leads to Clearance of Autoreactive Lymphocytes and Normalizes Immune Response in Lupus-Prone Mice. Front Immunol.

[110] Horwitz DA, Bickerton S, Koss M (2019). Suppression of Murine Lupus by CD4+ and CD8+ Treg Cells Induced by T Cell-Targeted Nanoparticles Loaded With Interleukin-2 and Transforming Growth Factor β. Arthritis Rheumatol.

[111] Otomo K, Koga T, Mizui M (2015). Cutting Edge: Nanogel-Based Delivery of an Inhibitor of CaMK4 to CD4+ T Cells Suppresses Experimental Autoimmune Encephalomyelitis and Lupus-like Disease in Mice. J Immunol.

[112] Maeda K, Otomo K, Yoshida N (2018). CaMK4 compromises podocyte function in autoimmune and nonautoimmune kidney disease. J Clin Invest.

[113] Zhang J, Chen C, Fu H (2020). MicroRNA-125a-Loaded Polymeric Nanoparticles Alleviate Systemic Lupus Erythematosus by Restoring Effector/Regulatory T Cells Balance. ACS Nano.

[114] Heidegger S, Gössl D, Schmidt A (2016). Immune response to functionalized mesoporous silica nanoparticles for targeted drug delivery. Nanoscale.

[115] Wang Y, Zhao Q, Han N (2015). Mesoporous silica nanoparticles in drug delivery and biomedical applications. Nanomedicine (Lond).

[116] Xu C, Lei C, Yu C (2019). Mesoporous Silica Nanoparticles for Protein Protection and Delivery. Front Chem.

[117] Zhou Y, Quan G, Wu Q (2018). Mesoporous silica nanoparticles for drug and gene delivery. Acta Pharm Sin B.

[118] Look M, Saltzman WM, Craft J (2014). The nanomaterial-dependent modulation of dendritic cells and its potential influence on therapeutic immunosuppression in lupus. Biomaterials.

[119] Look M, Stern E, Wang QA (2013). Nanogel-based delivery of mycophenolic acid ameliorates systemic lupus erythematosus in mice. J Clin Invest.

[120] Song X, Gao J, Liu H (2021). Rapamycin alleviates renal damage in mice with systemic lupus erythematosus through improving immune response and function. Biomed Pharmacother.

[121] Li-fu Miao, Jing Yang, Chao-lian Huang (2008). [Rapamycinloaded poly (lactic-co-glycolic) acid nanoparticles for intraarterial local drug delivery: preparation, characterization, and in vitro/in vivo release]. Zhongguo Yi Xue Ke Xue Yuan Xue Bao.

[122] Holladay C, Power K, Sefton M (2011). Functionalized scaffold-mediated interleukin 10 gene delivery significantly improves survival rates of stem cells in vivo. Mol Ther.

[123] Amend A, Wickli N, Schäfer AL (2021). Dual Role of Interleukin-10 in Murine NZB/W F1 Lupus. Int J Mol Sci.

[124] Biswas S, Bieber K, Manz RA (2022). IL-10 revisited in systemic lupus erythematosus. Front Immunol.

